# Detection and Localisation of PrP^Sc^ in the Liver of Sheep Infected with Scrapie and Bovine Spongiform Encephalopathy

**DOI:** 10.1371/journal.pone.0019737

**Published:** 2011-05-12

**Authors:** Sally J. Everest, Andrew M. Ramsay, Melanie J. Chaplin, Sharon Everitt, Michael J. Stack, Michael H. Neale, Martin Jeffrey, S. Jo Moore, Susan J. Bellworthy, Linda A. Terry

**Affiliations:** 1 Department of Pathology and Host Susceptibility, Veterinary Laboratories Agency, New Haw, United Kingdom; 2 Department of Pathology and Host Susceptibility, Veterinary Laboratories Agency (VLA-Lasswade), Penicuik, Midlothian, United Kingdom; Creighton University, United States of America

## Abstract

Prions are largely contained within the nervous and lymphoid tissue of transmissible spongiform encephalopathy (TSE) infected animals. However, following advances in diagnostic sensitivity, PrP^Sc^, a marker for prion disease, can now be located in a wide range of viscera and body fluids including muscle, saliva, blood, urine and milk, raising concerns that exposure to these materials could contribute to the spread of disease in humans and animals. Previously we demonstrated low levels of infectivity in the liver of sheep experimentally challenged with bovine spongiform encephalopathy. In this study we show that PrP^Sc^ accumulated in the liver of 89% of sheep naturally infected with scrapie and 100% of sheep challenged with BSE, at both clinical and preclinical stages of the disease. PrP^Sc^ was demonstrated in the absence of obvious inflammatory foci and was restricted to isolated resident cells, most likely Kupffer cells.

## Introduction

Transmissible spongiform encephalopathies (TSEs) or prion diseases are a group of fatal neurodegenerative disorders which include Creutzfeldt–Jakob disease (CJD) and Kuru in humans, bovine spongiform encephalopathy (BSE) in cattle, scrapie in sheep and goats and chronic wasting disease (CWD) in deer. The causative agent is widely believed to be a misfolded isoform of the normal, ubiquitously expressed cellular protein, PrP^c^, that converts to the pathogenic form (PrP^Sc^) and accumulates predominantly in the central nervous system [1].

Scrapie is endemic in the sheep populations of many countries throughout the world and has been recognised as a disease entity for almost 300 years. Sheep scrapie is not directly linked with human TSEs, although it has been implicated as a possible putative source of BSE in cattle [2] and remains a natural reservoir of TSE infection. Since the BSE epidemic, and particularly since the emergence of variant CJD, scrapie has been more closely monitored because of the possibility that BSE was transmitted to sheep [3] via exposure to the same infected feed that disseminated BSE to cattle. Given the widespread distribution of scrapie infectivity throughout the ovine carcase [4], the potential risk to human health of BSE in sheep could be much greater than BSE in cattle, where there is a more restricted tissue involvement [5], [6].

Restrictions on the use of sheep carcases for human consumption currently excludes the skull, together with the brain and eyes, tonsils and spinal cord of animals over 12 months old and the spleens and ileum of all sheep (defined by EC Regulation 999/2001 (Annex V), and enforced in England through Transmissible Spongiform Encephalopathies (England) Regulations 2010), put in place specifically to minimise the risk to human health from putative BSE infection of sheep. However, sheep offal is not excluded. Previously we demonstrated low levels of infectivity in the liver of sheep experimentally infected with BSE [7]. Here we demonstrate biochemical detection of PrP^Sc^ in the liver of the majority of sheep tested that were infected with scrapie or BSE.

## Results

### Detection of PrP^Sc^ in the liver of sheep naturally infected with scrapie

**Figure 1:**
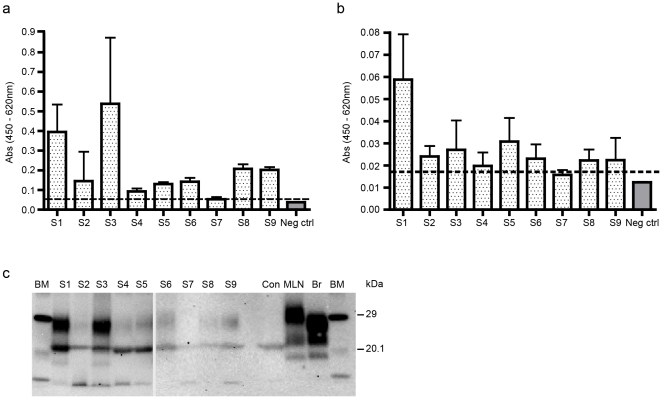
Detection of PrP^Sc^ in the livers of VRQ/VRQ scrapie-infected ewes. Liver samples were taken from 9 (S1–S9) VRQ/VRQ scrapie-infected ewes and 15 unexposed sheep (neg ctrl) and analysed for PrP^Sc^ using (a) the IDEXX Herdchek assay (b) the Bio-Rad TeSeE ELISA and (c) and the Bio-Rad TeSeE Sheep and Goat Western blot. Values are shown as the mean and standard deviations (n = 4 or 6). The cut-off points for the assays are shown (dotted line). BM  =  biotinylated markers with molecular mass shown in kDa. Mesenteric lymph node (MLN) and brain (Br) from confirmed scrapie positive sheep are shown as positive controls. Liver from a negative sheep (Con) is also shown.

The presence of PrP^Sc^ in the liver of nine sheep exposed to scrapie was assessed using modified versions of the IDEXX HerdChek BSE-Scrapie Antigen Test Kit EIA ®, the Bio-Rad TeSeE Sheep and Goat EIA® and the Bio-Rad TeSeE® Western blot (WB) (Figure 1). All sheep were homozygous VRQ/VRQ (at residues 136, 154, 171 of the prion protein), obtained from a single flock and were aged between 20 and 30 months. Seven of the nine sheep were showing clinical signs of disease at the time of post mortem (Table 1) and all were confirmed scrapie positive following assessment of tissue from the central nervous system and the lymphoreticular system.

**Table 1:** Details of sheep used in the study.

Sample number	Genotype	Experimental status	Age(months)	Clinical signs	Lymphoid tissue
S1	VRQ/VRQ	Natural scrapie	21	None observed	Positive^a^
S2			21	None observed	Positive^a^
S3			30	Positive	Positive^a^
S4			20	Positive	Positive^a^
S5			20	Positive	Positive^a^
S6			22	Positive	Positive^a^
S7			22	Positive	Positive^a^
S8			24	Positive	Positive^a^
S9			22	Positive	Positive^a^
B1	ARQ/ARQ	BSEChallenged(Oral challenge at 6 months of age)	28	None observed	Positive^b^
B2			27	None observed	Positive^b^
B3			27	None observed	Positive^b^
B4			27	None observed	Positive^b^
B5			29	Early positive	Positive^b^
B6			30	Early positive	Positive^b^
B7			33	Early positive	not tested

Positive^a^ represents a positive lymphoid tissue result when assayed by EIA and positive^b^ is a positive lymphoid tissue result by IHC. All animals were positive for PrP^Sc^ in brain.

Eight of the nine liver samples gave values above the cut-off point determined from analysis of livers from scrapie negative control sheep, for both the IDEXX HerdChek assay (OD range 0.053–0.538) (Figure 1a) and the Bio-Rad EIA (OD range 0.015–0.059) (Figure 1b). Western blot analysis further confirmed the presence of PK resistant PrP^Sc^ bands (Figure 1c) in the eight livers positive by the two assays. A band of approximately 30 KDa was observed in the liver samples with a similar relative molecular mass to the di-glycosylated band of PrP^Sc^ observed in samples extracted from the brain from a scrapie positive sheep (Figure 1c Br). The intensity of this band varied between individual samples and mirrored the value obtained for the EIA. A second band was observed at approximately 22 KDa relative molecular mass which also varied in intensity between samples. However, this band was also observed in the negative control and is not likely to be specific for PrP^Sc^. A third band with a faster mobility and with a molecular mass of approximately 18 KDa was seen in two lanes corresponding to the samples where the intensity of the other bands was greatest. This band had a similar mobility to the unglycosylated polypeptide from samples extracted from brain. The PrP^Sc^ from the lymph node migrated differently to that from the brain, but the signature three polypeptide cleavage products were still apparent. This observation has been reported elsewhere and is likely to be a result of increased glycosylation [8], [9], [10].

PrP^Sc^ was not detected in livers from 15 VRQ/VRQ ewes, ranging in age from 44–93 months old, sampled from a scrapie-free flock (Figure 1, negative controls). Further analysis by Western blot confirmed that PrP^Sc^ could not be detected in the liver from unexposed sheep, examples of which are shown in figure1(c).

### PrP^Sc^ in the livers from BSE-infected sheep

**Figure 2:**
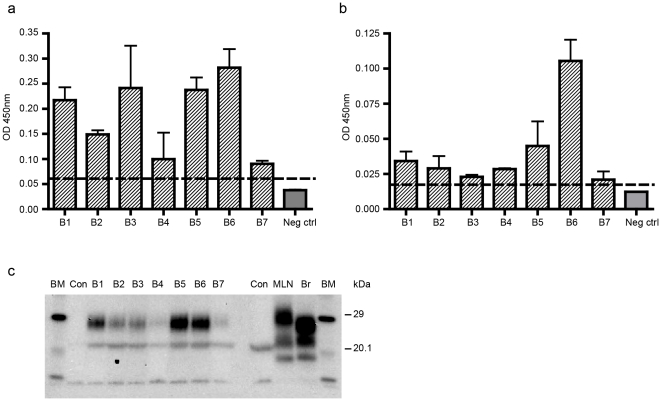
Detection of PrP^Sc^ in the livers of ARQ/ARQ BSE-challenged ewes. Liver samples from 7 (B1–B7) ARQ/ARQ BSE-challenged ewes and 15 negative control sheep (neg ctrl) were analysed for the presence of PrP^Sc^ using (a) the IDEXX Herdchek assay (b) the Bio-Rad TeSeE ELISA and (c) the Bio-Rad TeSeE sheep and goat Western blot. Values are shown as the mean and standard deviations (n = 4). The cut-off points of the assays are shown (dotted line). M  =  molecular mass markers (kDa). Mesenteric lymph node (MLN) and brain (Br) from confirmed scrapie positive sheep are shown as positive controls. Liver from a negative sheep (C) is also shown.

The presence of PrP^Sc^ in the liver of seven sheep challenged with BSE was assessed using the same diagnostic tests as above (Figure 2). All sheep were homozygous ARQ/ARQ (at residues 136, 154, 171) of the PrP protein and were aged between 27 and 33 months. Three of the seven sheep were showing early clinical signs of disease at the time of post mortem (Table 1) and all were confirmed positive following post mortem diagnostic investigation of tissue from the central nervous system and the lymphoreticular system.

All seven ARQ/ARQ sheep orally challenged with BSE gave values above the cut-off point for both the IDEXX HerdChek assay (OD range 0.090–0.282) (Figure 2a) and the the Bio-Rad EIA (OD range 0.021–0.106) (Figure 2b). Western blot analysis further confirmed the presence of PK resistant PrP^Sc^ in all seven liver samples (Figure 2c). These results are consistent with the previous demonstration that infectivity is present in the liver from BSE-infected sheep 16 months after challenge as shown by mouse bioassay [7].

### Localisation of PrP^Sc^ in isolated cells in the liver from scrapie infected and BSE-challenged ewes

**Figure 3:**
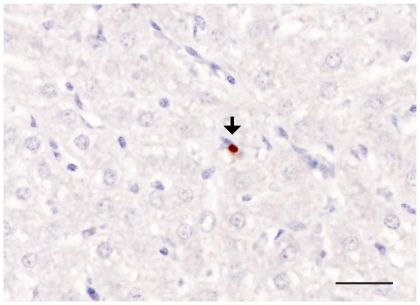
Detection of PrP^Sc^ deposits in the liver by IHC using mAb R145. Arrow head indicate PrP^Sc^ deposits in Kupffer cells of a naturally scrapie infected sheep. Scale bar  = 25 µm.

Immunohistochemistry (IHC), using anti-PrP monoclonal antibody R145, showed PrP^Sc^ depositions in liver samples from both the scrapie and BSE infected sheep. Similar IHC patterns of labelling were also observed with antibodies Bar224, P4 and BG4 (data not shown). Labelling of PrP^Sc^ was not observed associated with hepatocytes but was most commonly associated with cells with round or slightly flattened basophilic nuclei, putatively identified as Kupffer cells, which phagocytose spent red blood cells and other particulate debris from the blood (Figure 3). However, PrP^Sc^ association with the sinusoidal lining cells, which also have a phagocytic role, could not be ruled out. At least one section was examined for each animal and positive cells were detected in all animals examined, the maximum was 4–5 positive cells per section but more usually there was 1–3 cells. Detection of positive cells by IHC mirrored the results seen by both EIA and Western blot. The absence of obvious inflammatory leucocyte infiltrates in the vicinity of the PrP^Sc^ labelled cells indicated that the presence of prion protein was not likely to be due to localised inflammation.

## Discussion

In this study, the presence of PrP^Sc^ in the liver of sheep, infected with either scrapie or BSE, was demonstrated by EIA, Western blot and IHC. Furthermore, PrP^Sc^ was detected in the livers of TSE infected sheep in the presence and absence of clinical signs. The PrP^Sc^ was localised to isolated cells lining sinusoids that are most likely to be Kupffer cells. Confirmation of the presence of PrP^Sc^ in the liver is consistent with our previous study demonstrating transmission of infectivity from liver of BSE challenged sheep to mice [7].

In previous studies PrP^Sc^ was not detected, by IHC, in the liver at any stage during BSE infection of orally infected Texel sheep [11] nor has it been detected in sheep following challenge with BSE by the intracranial route (Martin Jeffrey, unpublished observation). The reason for the presence of PrP^Sc^ in the liver of some infected sheep and not others is uncertain but host genotype, agent strain/source, dose and route of challenge may all be influential.

One possible mechanism for extra-neuronal and extra-lymphatic deposition of prions may be linked to the secondary inflammatory conditions of organs, leading to the development of isolated lymphoid follicles [12]. Using rodent models of chronic inflammatory disease, studies have shown that prion accumulation can occur in otherwise prion-free organs including the kidney, liver and pancreas [12]. These observations have been extended to the mammary gland of sheep [13]. However, the distribution and pattern of labelling in isolated cells in the liver sinuses is not consistent with this mechanism. Indeed others have demonstrated prions in the kidneys of sheep in the absence of inflammatory conditions and propose that accumulation in the kidney results from direct exposure to prions in blood [14]. This might suggest similar mechanisms of PrP^Sc^ accumulation in liver given that scrapie and BSE- affected sheep accumulate infectivity in blood during both the preclinical and clinical stages of the disease [15], [16]. Furthermore, liver is directly supplied by blood leaving the alimentary canal, a source of prions even at very early stages of natural and experimental scrapie infection in sheep [17], [18], [19] In the present study PrP^Sc^ labelling was observed only in what were putatively identified as Kupffer cells. A major function of Kupffer cells is to clear particulate substances from the blood and this is consistent with the proposed blood-borne mechanism of accumulation.

Kupffer cells were labelled with all PrP specific antibodies tested including BG4 which recognises the extreme N terminus of PrP (data not shown).This indicates that the PrP^Sc^ in Kupffer cells is the full length protein and is probably not internalised to lysosomal compartments. This might indicate distinct trafficking pathways of prions in this cell type compared with those of neurons, glia and macrophages, in which the prion protein is mostly truncated or it may represent sequestered PrP^Sc^ which does not undergo translocation to lysosomes.

Sequestration of PrP^Sc^ in the liver is not likely to contribute to the natural spread of the disease between sheep although it could pose a threat to food safety if a zoonotic prion disease, such as BSE, were identified in the sheep flock. Specified risk material (SRM) controls are in place across the EU as a precautionary measure to reduce the risk of BSE-infected meat from sheep from entering the food chain. SRM in sheep includes the skull, together with the brain and eyes, tonsils and spinal cord of animals over 12 months old and the spleens and ileum of all sheep. The SRM controls require that these parts of the sheep are removed and destroyed to reduce the theoretical risk of human exposure to a putative zoonotic infection: it is estimated that the SRM are responsible for about a third of the possible carcase infectivity. Sheep offal, however, is not included in these controls and is consumed from sheep of all ages. However, it should be highlighted that on-going targeted and scanning surveillance for sheep and goat TSEs in EU member states has so far not identified any BSE in sheep [20], [21]. Thus it is perceived that the risk to human health from possible infection of sheep with BSE is low (Spongiform Encephalopathy Advisory Committee statement 2008).

The finding that PrP^Sc^ is sequestered in the liver of an unexpectedly high percentage of VRQ/VRQ sheep naturally infected with scrapie and of ARQ/ARQ sheep experimentally challenged with BSE provides further information on the organ distribution of infectivity in the host. It will also be important to determine whether host species, genotype and prion strain impact on prion distribution in extra-neural and extra-lymphatic tissues using more sensitive diagnostic methods.

## Materials and Methods

### Animals and tissues

All experiments involving animals carried out within VLA are supervised by a named Veterinary surgeon as required under UK legislation and individual experiments are approved by UK government Home office inspectors (Home Office Project licenses: PPL70/5782 and PPL70/4495).

Liver samples from nine scrapie infected sheep bearing the PrP genotype, VRQ/VRQ (at residues 136, 154 and 171 respectively) were taken at post mortem. The sheep used in these experiments originated from a flock where scrapie is endemic, resulting in the natural infection of 100% of sheep carrying either the VRQ/VRQ or ARQ/VRQ PrP genotypes.

Scrapie infection was demonstrated by the detection of PrP^Sc^ in the brain by immunohistochemistry (IHC) and by biochemical analysis; seven of the nine ewes were showing clinical signs of scrapie at the time of post mortem (Table 1). The sheep ranged in age from 20 to 30 months and were of mixed breeds; three Welsh Mountain, two Cheviots and four Polled Dorset/Friesland crosses.

In addition, seven liver samples from ARQ/ARQ Suffolk sheep, ranging in age from 27 to 33 months of age, orally challenged with 5 g UK BSE-infected bovine brain tissue, were investigated. Three of these ewes were showing early clinical signs at the time of death but all were infected with BSE as demonstrated by the presence of PrP^Sc^ in the brain (Table 1). All sheep were also examined for PrP^Sc^ presence in lymphoid tissue either by IHC or Western blot.

Negative control liver samples (n = 15) were also taken from sheep not previously exposed to scrapie. These were obtained from a New Zealand derived scrapie-free flock maintained under strict bio-security. The sheep were age and genotype matched with the disease-affected sheep where possible. The sheep were confirmed negative for scrapie by IHC for the presence of PrP^Sc^ in the brain. All unfixed liver samples were stored at −80°C prior to analysis.

### ELISA and Western blot analysis

Two methods of on-plate immunoassay were used to detect PrP^Sc^ in the liver samples, the Bio-Rad TeSeE sheep and goat ELISA and the IDEXX herdchek scrapie/BSE antigen EIA. Sample extraction was carried out according to the manufacturer's instructions for the Bio-Rad TeSeE sheep and goat ELISA with several modifications. In brief, liver tissue was ribolysed to produce a 20% (w/v) homogenate and digested using Proteinase K. Following precipitation and centrifugation at 15,000 g for 7 minutes, the pellets were solubilised by incubating at 100°C for 5 minutes in the buffer provided. The reconstituted pellets were applied to the microtitre plates coated with the first anti-PrP antibody and the plate reacted for 2 hours at room temperature. After washing, enzyme conjugate was added and plates were left for 2 hours at room temperature, the plate was then washed and 100 µl tetramethyl benzidine (TMB) substrate was added for 30 minutes at room temperature in the dark. The reaction was blocked and the absorbance read at 450 nm and 620 nm.

The “IDEXX HerdChek scrapie/BSE Antigen EIA Test”, was carried out according to the manufacturer's instructions with some modifications. The liver samples were homogenised as described but with the incorporation of a single large (6 mm diameter) ceramic bead and three agitation cycles at 6.5 rpm each for 45 seconds. The homogenates were cooled between each cycle, mixed with the working plate diluents and added to the 96-well plate provided. The plate was agitated for 2.5 hours at room temperature, washed six times with wash solution 1, a conditioning buffer was added to each well for 10 minutes and the plate again washed for a further 3 cycles. Immobilised PrP^Sc^ on the plate was detected by incubation with a peroxidase conjugated anti-PrP antibody and was visualised with TMB substrate. Following incubation for 15 minutes, the reaction was quenched by the addition of 1M hydrochloric acid and the absorbance was read at 450 nm and 620 nm.

### Setting of threshold values for adapted rapid tests

Negative control livers from 15 VRQ/VRQ or ARQ/ARQ ewes, ranging in age from 44–93 months old, sampled from a scrapie-free flock were assayed using the IDEXX HerdChek test and Bio-Rad TeSeE EIA, using the modifications described above. These data were used to determine cut-off values for positive signals. The diagnostic tests have cut-off values that are set by the manufacturers for brain but in this study cut-off values were determined to take account of the method modifications and the different tissue type analysed. Thresholds of 3 standard deviations above the mean were calculated and values of 0.017 and 0.059 absorbance values were set for the Bio-Rad TeSeE and IDEXX Herdchek EIAs respectively. These values are lower than those set by the manufacturer for brain.

For the Western blot analysis sample extraction was carried out according to the manufacturer's instructions (Bio-Rad TeSeE Western Blot). Frozen liver tissue was thawed and ribolysed to give a 20% (w/v) homogenate. The samples were digested with Proteinase K and the reaction stopped with kit reagent B. Following precipitation and centrifugation at 15,000 *g* for 7 minutes, in accordance with the Bio-Rad TeSeE Western blot protocol, the pellets were re-suspended in Laemmli sample buffer, heated at 100°C for 5 minutes and centrifuged at 15,000 g for 15 minutes.

For analysis, 15 µl from each sample were loaded on a 12% Criterion XT Bis-tris SDS gel (Bio-Rad) and subjected to electrophoresis in XT-MOPS running buffer (Bio-Rad) at 200 V for 50 minutes. Proteins were transferred to a PVDF membrane (Bio-Rad) at 115 V, 60 min using Tris/CAPS transfer buffer (Bio-Rad).

The membranes were incubated for 30 minutes with the kit blocking solution before incubation for one hour with the primary PrP antibody, SHA31, and then with goat anti-mouse IgG antibody conjugated to horseradish peroxidase (Bio-Rad) prior to visualization by chemiluminescence (ECL; Amersham). The signal was captured using a Bio-Rad Fluor-S multi-imager.

### Immunohistochemistry

Post-mortem liver samples were placed in formal saline for a minimum of three days before processing and embedding in paraffin wax. IHC detection of PrP^Sc^ was performed using rat MAb R145, raised against a synthetic peptide and specific for residues 221–233 of bovine PrP (YQRESQA YYQRGA), Bar224 (141–147 of ovine PrP), P4(93–97 of ovine PrP) and BG4 (46–54 of Bovine PrP). Sections of the paraffin embedded tissues were cut at 4 µm onto charged slides and incubated at 60°C for 30 minutes to improve adhesion. Sections were de-waxed in xylene, and rehydrated. Epitope demasking was performed by immersion of sections for 30 minutes in undiluted formic acid, washing in running tap water for 15 minutes, followed by autoclaving at 121°C in citrate buffer pH 6.1 (8.8 mM sodium citrate dihydrate, 1.3 mM citric acid in 2 litres purified water). Endogenous peroxidase was blocked using 3% hydrogen peroxide (100 w/vol) in methanol, and the washing buffer used throughout the procedure was tris buffered saline, supplemented with 0.2% tween20 (TBST). R145 was applied at 2 µg/ml for one hour at room temperature, with immunodetection performed using biotinylated rabbit anti-rat IgG (Vector Laboratories) and the avidin-biotin-peroxidase-complex (Vector Elite) technique using diaminobenzidine chromogen prepared in McIlvane's citrate buffer. Sections were counterstained using Mayer's haematoxylin, then dehydrated, cleared and mounted in dibutylphthalate in xylene (DPX), before examination by light microscopy.
